# Bioequivalence of Fixed-Dose Dapagliflozin-Pioglitazone in Healthy Indian Adults: Results From the Randomized Crossover PRO-4 Study

**DOI:** 10.7759/cureus.111872

**Published:** 2026-07-01

**Authors:** J. Hari Prasath, Umesh Garg, Kalyan Kurapati, Abhamoni Baro Agarwal, Parul Singhal, Thamburaj Anthuvan, Smriti Gadia, Amit Gupta, Sridhar S B.

**Affiliations:** 1 Internal Medicine, Apollo First Med Hospitals, Chennai, IND; 2 Endocrinology and Diabetes, Pushpanjali Hospital and Research Centre, Agra, IND; 3 Cardiology, Shalini Heart Hospital, Nizamabad, IND; 4 Endocrinology, Arya Wellness Centre, Guwahati, IND; 5 Endocrinology, Dispur Polyclinic and Hospitals, Guwahati, IND; 6 Endocrinology and Diabetes, Singhal Clinic, Kashipur, IND; 7 Sales and Marketing, USV Private Limited, Mumbai, IND; 8 Scientific Services, USV Private Limited, Mumbai, IND; 9 Clinical Research, USV Private Limited, Mumbai, IND

**Keywords:** bioequivalence, crossover study, dapagliflozin, fixed-dose combination, pioglitazone

## Abstract

Objective

The study assessed the bioequivalence of a fixed-dose combination (FDC) of dapagliflozin 10 mg/pioglitazone 15 mg co-administered with the reference products Forxiga® 10 mg: AstraZeneca Pharmaceuticals LP, Indiana, USA and Pioglit® 15 mg: Sun Pharma Laboratories Ltd., Assam, India in healthy adult participants under fasting conditions.

Materials and methods

A randomized, open-label, single-dose, two-period, crossover design was used in the study. Eligible participants were randomly assigned to receive either the test FDC or the respective reference products during the first period, followed by the administration of the alternate treatment after a 7-day washout interval. The primary pharmacokinetic parameters included maximum plasma concentration (C_max_) and area under the plasma concentration-time curve from time zero to last measurable concentration (AUC_0_-t), while secondary parameters were area under the curve extrapolated to infinity (AUC_0_-∞), time to reach maximum plasma concentration (T_max_), and elimination half-life (t½).

Results

Forty-two healthy male volunteers were randomized; 39 completed both study periods. For dapagliflozin, the geometric mean of the test-to-reference ratios were 94.22% for C_max_ (90% CI: 86.54%-102.58%) and 98.04% for AUC_0_-t (90% CI: 96.01%-100.11%). For pioglitazone, the corresponding values were 102.35% for C_max_ (90% CI: 88.12%-118.87%) and 107.39% for AUC_0_-t (90% CI: 92.97%-124.05%). All results were within the acceptable bioequivalence range of 80.00-125.00%. Two mild adverse events were observed, with no serious or clinically significant safety issues reported.

Conclusion

The FDC of dapagliflozin and pioglitazone was bioequivalent to the individual reference products in fasting, healthy male subjects and was well-tolerated.

## Introduction

Type 2 diabetes (T2D) is a progressive condition marked by increasing insulin resistance and declining beta-cell function, often leading to a loss of glycemic control over time. As a result, many patients do not reach or maintain their glycemic targets with monotherapy alone and require combination therapy with agents that target different underlying pathophysiological mechanisms [1,2]. In this context, fixed-dose combinations (FDCs) can offer therapeutic and practical benefits by reducing pill burden and supporting long-term management adherence.

Dapagliflozin, a sodium glucose cotransporter 2 (SGLT2) inhibitor, effectively lowers plasma glucose levels through an insulin-independent mechanism by inhibiting renal glucose reabsorption and promoting urinary glucose excretion. This pharmacological action enables efficacy across various stages of T2D, including in patients with progressive beta-cell dysfunction. The drug is generally well tolerated, carries a low intrinsic risk of hypoglycemia, and is commonly used in combination therapy [3].

Pioglitazone, a thiazolidinedione (TZD), enhances insulin sensitivity through activation of peroxisome proliferator-activated receptor gamma (PPAR-γ) [4]. By targeting insulin resistance, a central abnormality in T2D, pioglitazone complements the insulin-independent mechanism of dapagliflozin. It remains a clinically relevant oral antidiabetic agent, particularly in patients in whom improving insulin sensitivity is desirable.

Therefore, the combination of dapagliflozin and pioglitazone has a strong pharmacological rationale, with complementary mechanisms that support their combined use in patients requiring more than one oral antidiabetic agent. Emerging clinical evidence suggests that this combination is effective and well tolerated in patients with inadequately controlled T2D, and broader data support the use of pioglitazone in combination with SGLT2 inhibitors in routine clinical practice. The development of an FDC of these agents may further enhance treatment convenience and adherence by simplifying the therapy. 

For a newly developed FDC, demonstrating bioequivalence to co-administered individual reference products is required to establish pharmacokinetic comparability and support therapeutic equivalence. Single-dose, randomized crossover studies in healthy volunteers are the standard approach for these evaluations [5,6]. Accordingly, this study was conducted to assess the bioequivalence of the dapagliflozin and pioglitazone FDC. 

This study is part of the Pioglitazone-SGLT2 Inhibitor Combination Research Outcomes (PRO) program, a structured research framework designed to evaluate the clinical, evidence-based, and pharmacokinetic aspects of the dapagliflozin-pioglitazone combination. Within this program, PRO-1 evaluated clinical efficacy and safety [7], while PRO-2 and PRO-3 provided a systematic review and an expert clinical perspective, respectively. The present study (PRO-4) evaluates the bioequivalence of an FDC containing dapagliflozin and pioglitazone (10 mg/15 mg) relative to the corresponding individual reference products.

The primary objective of the study was to compare the rate and extent of absorption of the fixed-dose combination of dapagliflozin and pioglitazone 10 mg/15 mg tablet (test product) with those of the corresponding reference products in healthy adult subjects under fasting conditions to assess bioequivalence. The secondary objective of the study was to evaluate the safety and tolerability of a single oral dose of the investigational product.

## Materials and methods

Study design 

This study was designed as a randomized, open-label, single-dose, two-treatment, two-period, two-sequence crossover oral bioequivalence trial conducted under fasting conditions in healthy adult male volunteers. Eligible participants were randomized to treatment sequences using a predefined randomization schedule generated with PROC PLAN in SAS® version 9.4 (SAS Institute, Cary, USA), with block randomization to ensure balanced treatment allocation. Participants were evenly divided into test-reference (TR) and reference-test (RT) sequences, with each receiving both the test (T) and reference (R) formulations across two study periods, as scheduled. Allocation followed the pre-generated plan, ensuring balanced and unbiased assignment. Although the study was open-label, bioanalytical staff remained blinded to treatment assignment (the sequence of test and reference formulations) until sample analysis was complete, reducing potential analytical bias.

Each dosing period was separated by a 7-day washout, corresponding to at least five elimination half-lives of the study drugs. Based on the known elimination half-lives of dapagliflozin and pioglitazone, this interval was considered sufficient to minimize the risk of residual drug concentrations before the subsequent dosing period.

The study followed Central Drugs Standard Control Organization (CDSCO) guidelines for bioavailability and bioequivalence, as well as the Declaration of Helsinki, International Council for Harmonisation (ICH) Good Clinical Practice (GCP) standards, Indian Council of Medical Research (ICMR) Ethical Guidelines for Biomedical Research on Human Participants (2017), and the New Drugs and Clinical Trials Rules, 2019. The Life Point Research Ethics Committee approved the protocol and informed consent forms on 01 March 2024. Conducted under the supervision of Principal Investigator Dr. Abdul Indikar and with ethics committee approval, the study ensured that all participants provided written informed consent before their participation.

The study was not prospectively registered with the Clinical Trials Registry of India, a limitation acknowledged. However, it was conducted with ethics committee approval and in accordance with applicable regulatory standards for bioavailability and bioequivalence studies.

Study participants

Healthy adult male volunteers aged 18-45 years with a BMI between 18.0 and 30.0 kg/m² were included in the study. Only male subjects were selected to minimize potential variation in pharmacokinetics due to hormonal changes, particularly those occurring during the menstrual period. Health status was confirmed through physical examination, medical history, ECG, vital signs, chest radiography, and routine laboratory investigations. The main exclusion criteria included known hypersensitivity to dapagliflozin, pioglitazone, or any excipients in the study products; clinically significant medical conditions; abnormal renal function, including a clinically relevant reduction in creatinine clearance; a history or presence of alcohol or drug abuse; major illness within 90 days before screening; and blood donation or transfusion within 90 days before study dosing.

Sample size estimation

Sample size was determined considering the expected intra-subject variability of key pharmacokinetic parameters and the goal of demonstrating bioequivalence between the products. Bioequivalence was established if the 90% confidence intervals (CIs) for the geometric least-squares mean (LSM) ratios (test/reference) of peak plasma concentration (C_max_) and the area under the plasma concentration-time curve up to the last quantifiable time point (AUC_0_-t) were within 80.00% to 125.00%, at a significance level (α) of 0.05, with at least 80% power. Based on these criteria, 36 evaluable subjects were deemed sufficient to confirm bioequivalence under fasting conditions. To account for potential dropouts, withdrawals, or noncompliance, 42 subjects were enrolled and received the study drug.

Study treatments and dosing 

The test treatment was an FDC tablet containing dapagliflozin 10 mg and pioglitazone 15 mg manufactured by USV Private Ltd., Mumbai, India. The reference treatment consisted of the coadministration of individual reference products: dapagliflozin 10 mg (Forxiga®, AstraZeneca Pharmaceuticals LP, Indiana, USA) and pioglitazone 15 mg (Pioglit®: Sun Pharma Laboratories Ltd., Assam, India). 

In each study period, after a minimum fasting period of 10 h, participants received either the test FDC tablet or the reference treatment according to the randomization schedule. The study medication was administered in approximately 240 mL of a 20% aqueous glucose solution. To ensure standardized carbohydrate supplementation and minimize the risk of hypoglycemia, the participants received 60 mL of 20% aqueous glucose solution at 15-minute intervals for 4 h after dosing. The same glucose administration schedule was applied in both treatment periods and to both the test and reference treatments, thereby minimizing the potential for differential effects on the comparative pharmacokinetic assessment. Water intake was restricted from 1 h before dosing to 1 h after dosing, except for protocol-specified glucose administration.

The clinical phase spanned 11 days and included both the dosing periods and the washout interval. Subjects remained confined to the clinical facility until completion of the 24-hour post-dose blood sample in each period, after which the 36- and 48-hour samples were collected on an outpatient basis.

Blood sampling and sample handling

For pharmacokinetic assessment, blood samples were collected at multiple time points: pre-dose (0 hours) and at 0.17, 0.33, 0.50, 0.75, 1.00, 1.33, 1.67, 2.00, 2.33, 2.67, 3.00, 3.33, 3.67, 4.00, 4.50, 5.00, 6.00, 7.00, 8.00, 12.00, 16.00, 24.00, 36.00, and 48.00 hours after dosing in each study period. Blood was drawn into pre-labeled dipotassium ethylenediaminetetraacetic acid (K2EDTA) vacutainers either via an indwelling cannula or by direct venipuncture. The plasma was separated by centrifugation at 3800 rpm for 10 min at 10 ± 2°C, then divided into two aliquots in pre-labeled polypropylene tubes and stored at −70°C ± 15°C until bioanalytical assay.

Bioanalytical method

The plasma levels of dapagliflozin and pioglitazone were measured using a validated liquid chromatography-tandem mass spectrometry (LC-MS/MS) technique at Synergen Bio Pvt. Ltd., Pune, India. The method was validated before analyzing the study samples, in accordance with regulatory standards (US FDA/European Medicines Agency (EMA)), for selectivity, sensitivity, accuracy, precision, recovery, matrix effects, and stability.

Pharmacokinetic analysis

The primary pharmacokinetic parameters included C_max_ and AUC_0_-t. The secondary pharmacokinetic parameters included the area under the curve extrapolated to infinity (AUC_0_-∞), time to maximum plasma concentration (T_max_), terminal elimination half-life (t½), elimination rate constant (Kel), and percentage of AUC extrapolated beyond the last measurable concentration (AUC%Extrap).

Statistical analysis

The validated SAS® (version 9.4) methods, in accordance with regulatory guidelines, were used for bioequivalence studies, ensuring the accuracy and reproducibility of the results. Descriptive statistics were calculated for all pharmacokinetic parameters. Bioequivalence was evaluated using the log (ln)-transformed data of the main pharmacokinetic parameters for dapagliflozin and pioglitazone. An ANOVA model was employed, with treatment, period, and sequence as fixed factors, and subjects nested within sequence as a random factor. The effects of period and formulation were tested at a 5% significance level. The significance of the sequence effect was assessed using a model with subjects nested within sequence, also at the 5% level.

Geometric LSM ratios (test/reference) and their 90% CIs were derived by back-transforming the ln-transformed data to the original scale. In line with CDSCO bioavailability and bioequivalence guidance, as well as internationally accepted FDA and EMA standards, bioequivalence was deemed proven if the 90% CIs for the test/reference geometric mean ratios of C_max_ and AUC_0_-t were within the range of 80.00% to 125.00%. The pharmacokinetic analysis population included those who completed both study periods and provided sufficient concentration-time data for parameter calculation. The safety analysis population included all participants who received at least one dose of the study medication.

Safety outcomes

A post-study laboratory safety sample was collected from all participants who received the dose. Safety was evaluated using clinical and laboratory monitoring. Assessments included hematology and biochemistry (pre- and post-study), chest X-ray (posteroanterior view), electrocardiogram (ECG), physical examination, and measurement of vital signs (body temperature, pulse rate, sitting blood pressure, and respiratory rate).

## Results

Subject disposition and demographics

**Figure 1:**
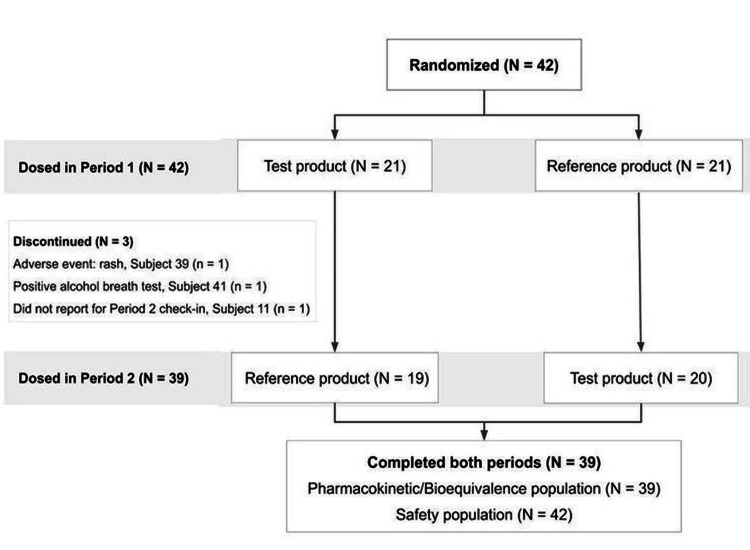
Subject disposition flow chart

Subject disposition details are shown in Figure 1. Forty-two healthy adult male participants were enrolled and randomized in this study. All received the study medication during Period 1, and 39 continued into Period 2. Three participants discontinued before Period 2: one (Participant No. 39) was withdrawn after Period 1 due to an adverse event (rash, possibly related to the reference treatment); another (Participant No. 41) was withdrawn before Period 2 for failing the breath alcohol test; and a third (Participant No. 11) missed the Period 2 screening visit and was dropped. Thirty-nine subjects completed both study periods, provided evaluable pharmacokinetic data for both drugs, and were therefore included in the pharmacokinetic and bioequivalence analysis population. The safety population included all forty-two subjects who received at least one dose of the investigational drug. No significant protocol deviations affecting safety, pharmacokinetics, or bioequivalence were reported.

All enrolled participants were Indian males. The mean ± standard deviation (SD) age was 31.1 ± 6.35 years, the mean body weight was 68.7 ± 9.76 kg, the mean height was 167.8 ± 5.52 cm, and the mean BMI was 24.35 ± 2.80 kg/m² (Table 1).

**Table 1:** Demographic and baseline characteristics (safety population, N = 42) Abbreviations: BMI, body mass index; SD, standard deviation.

Variable	Mean ± SD	Range
Age, years	31.1 ± 6.35	18–44
Weight, kg	68.7 ± 9.76	54.90–91.20
Height, cm	167.8 ± 5.52	157–178
BMI, kg/m²	24.35 ± 2.80	18.59–29.78

Dapagliflozin pharmacokinetic results

**Figure 2:**
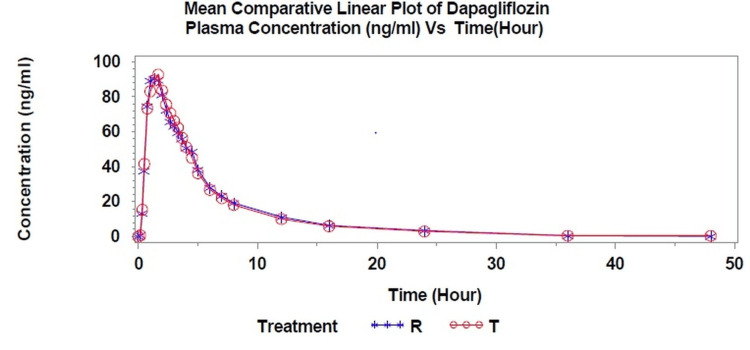
Mean plasma concentration–time profile of dapagliflozin (linear scale) Abbreviations: T, test; R, reference.

The plasma concentration-time profiles of the test and reference dapagliflozin under fasting conditions are presented in Figure 2. No statistically significant effects of period, sequence, or formulation were observed for the primary pharmacokinetic parameters (p > 0.05).

The geometric LSM ratios of the test/reference products were 94.22% for C_max_ and 98.04% for AUC_0_-t. The corresponding 90% CIs were 86.54%-102.58% for C_max_ and 96.01%-100.11% for AUC_0_-t (Table 2). The intra-subject coefficient of variation was 22.51% for C_max_ and 5.47% for AUC_0_-t, respectively. All 90% CIs fell within the pre-specified bioequivalence range of 80.00%-125.00%.

**Table 2:** Primary pharmacokinetic parameters of dapagliflozin (PK population) Abbreviations: AUC_0_–t, area under the plasma concentration–time curve from time zero to last measurable concentration; C_max_, maximum plasma concentration; CI, confidence interval; CV, coefficient of variation; GMR, geometric mean ratio; PK, pharmacokinetic.

Parameter	Test (N = 20)	Reference (N = 19)	GMR (%)	90% CI (%)	Intra-subject CV (%)
C_max_ (ng/mL)	103.50	109.86	94.22	86.54–102.58	22.51
AUC_0_–t (ng·h/mL)	502.32	512.36	98.04	96.01–100.11	5.47

The secondary pharmacokinetic parameters are listed in Table 3. The median T_max_ was 1.67 h for both treatments. The mean t½ was 7.39 h for the test and 7.00 h for the reference product. The mean AUC_0_-∞ values were 559.63 and 554.01 ng·h/mL for the test and reference products, respectively.

**Table 3:** Secondary pharmacokinetic parameters of dapagliflozin (PK population) Note: Data are presented as mean (standard deviation), unless otherwise specified. Abbreviations: AUC%Extrap, extrapolated area under the curve; CV, coefficient of variation; Kel, Elimination rate constant; PK, pharmacokinetic; T_max_, time to reach maximum plasma concentration; t½, elimination half-life; AUC_0_-∞ , area under the curve extrapolated to infinity

Parameter	Test (N = 20)	CV (%)	Reference (N = 19)	CV (%)
T_max_ (h), median (range)	1.67 (0.5–4.5)	54.35	1.67 (0.5–4.5)	58.14
t½ (h)	7.39 (3.05)	41.22	7.00 (3.09)	44.22
AUC_0_–∞ (ng·h/mL)	559.63 (178.56)	31.90	554.01 (136.01)	24.55
AUC%Extrap	5.41 (6.51)	120.20	4.28 (1.72)	40.30
Kel (h⁻¹)	0.10 (0.042)	39.42	0.11 (0.040)	35.90

Pioglitazone pharmacokinetic results

**Figure 3:**
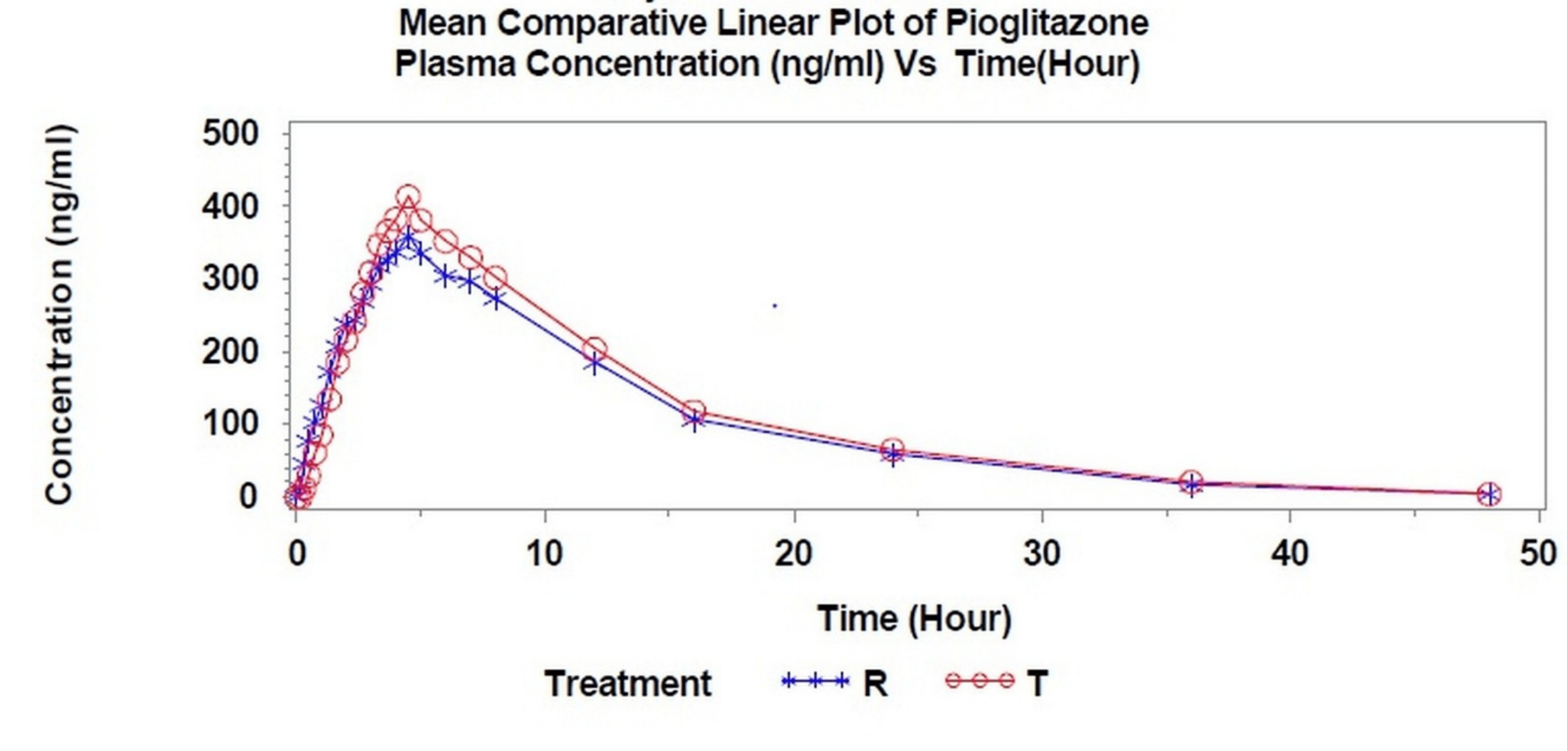
Mean plasma concentration–time profile of pioglitazone (linear scale) Abbreviations: T, test; R, reference

The plasma concentration-time profiles of the test and reference pioglitazone under fasting conditions are shown in Figure 3. The period and sequence effects were found to be statistically non-significant for the primary pharmacokinetic (p > 0.05).

The geometric LSM ratios (test/reference) were 102.35% for C_max_ and 107.39% for AUC_0_-∞. The corresponding 90% CIs were 88.12%-118.87% for C_max_ and 92.97%-124.05% for AUC_0_-t (Table 4). The intra-subject coefficient of variation was 40.71% for C_max_ and 39.12% for AUC_0_-t. The 90% CIs were within the bioequivalence acceptance range of 80.00-125.00%.

**Table 4:** Primary pharmacokinetic parameters of pioglitazone (PK population) Abbreviations: AUC_0_–t, area under the plasma concentration–time curve from time zero to the last measurable concentration; C_max_, maximum plasma concentration; CI, confidence interval; CV, coefficient of variation; GMR, geometric mean ratio.

Parameter	Test (N = 20)	Reference (N = 19)	GMR (%)	90% CI (%)	Intra-subject CV (%)
C_max_ (ng/mL)	369.22	360.75	102.35	88.12–118.87	40.71
AUC_0_–t (ng·h/mL)	4373.35	4072.29	107.39	92.97–124.05	39.12

Table 5 summarizes the secondary pharmacokinetic parameters. The median T_max_ was 4.5 h for both treatments. The mean t½ was 7.67 h for the test product and 9.62 h for the reference. The mean AUC_0_-∞ values were 5703.28 ng·h/mL for the test and 4835.87 ng·h/mL for the reference.

**Table 5:** Secondary pharmacokinetic parameters of pioglitazone (PK population) Note: Data are presented as mean (standard deviation), unless otherwise specified. Abbreviations: AUC%Extrap, extrapolated area under the curve; CV, coefficient of variation; Kel, Elimination rate constant; PK, pharmacokinetic; T_max_, time to reach maximum plasma concentration; t½, elimination half-life; AUC_0_–∞, area under the curve extrapolated to infinity

Parameter	Test (N = 20)	CV (%)	Reference (N = 19)	CV (%)
T_max_ (h), median (range)	4.5 (3.33–8.00)	24.45	4.5 (0.17–8.00)	40.63
t½ (h)	7.67 (3.27)	42.68	9.62 (8.61)	89.53
AUC_0_–∞ (ng·h/mL)	5703.28 (2611.49)	45.78	4835.87 (2693.16)	55.69
AUC%Extrap	5.30 (3.65)	69.00	9.10 (10.16)	111.64
Kel (h⁻¹)	0.10 (0.038)	36.81	0.09 (0.039)	40.74

Safety and tolerability

There were no deaths, serious adverse events, or hypoglycemia episodes during the study. Only two adverse events were reported in two subjects, with one occurring after Period 01 dosing and the other identified during the post-study clinical laboratory safety assessment. 

Subject no. 39 developed an abdominal rash after receiving the reference product during Period 01. No symptoms like fever, headache, nausea, or vomiting were reported. The subject was advised to rest and kept under close observation. At follow-up evaluation, the subject was asymptomatic with normal vital signs. The adverse event was assessed as mild in severity and possibly related to the investigational product.

Subject No. 35 showed a decrease in hemoglobin levels from 12.2 g/dL at baseline to 10.8 g/dL during the post-study laboratory assessment. At follow-up, the subject remained clinically stable and asymptomatic, with normal vital signs. A repeat hemoglobin evaluation showed improvement to 11.7 g/dL. The event was considered mild, resolved, and probably unrelated to the investigational product.

No abnormalities were observed in vital signs, physical examination, or ECG findings in any other subject during the study.

## Discussion

In this randomized, single-dose, crossover study conducted under fasting conditions, the FDC of dapagliflozin and pioglitazone was demonstrated to be bioequivalent to the concomitant administration of individual reference products in healthy adult participants. Bioequivalence was established for both analytes, with comparable rates and extents of absorption, as reflected by the C_max_ and AUC across all prespecified comparisons. The similar median T_max_ further supports consistent absorption.

The differences in pharmacokinetic variability between the two analytes align with their known characteristics. Dapagliflozin showed low intra-subject variability, yielding narrow confidence intervals and precise exposure estimates. In contrast, pioglitazone showed higher intra-subject variability (~40% for both C_max_ and AUC_0_-t). This relatively high variability is consistent with the published literature, which reports moderate-to-high pharmacokinetic variability for pioglitazone. Several factors may contribute to this observation. Pioglitazone undergoes extensive hepatic metabolism, primarily via CYP2C8 and, to a lesser extent, CYP3A4, both of which are subject to inter- and intra-individual variability. Additionally, variability in gastrointestinal absorption and first-pass metabolism may influence peak exposure (C_max_), while differences in plasma protein binding and distribution kinetics may further contribute. In comparison, dapagliflozin typically exhibits lower intra-subject variability, reflecting its more predictable absorption and metabolic profile, with glucuronidation (UGT1A9) as the primary route of metabolism and minimal involvement of cytochrome P450 (CYP)** **enzymes. [8,9]. Despite this variability, the 90% confidence intervals for both primary pharmacokinetic parameters remained within the predefined bioequivalence limits (80.00-125.00%), supporting the bioequivalence and robustness of the FDC formulation.

The safety findings were limited and clinically unremarkable in this study. A low incidence of mild adverse events was observed, with no serious or life-threatening events or episodes of hypoglycemia. The tolerability profile was consistent with the known safety characteristics of dapagliflozin and pioglitazone following single-dose administration in healthy subjects. 

Administering a 20% aqueous glucose solution after dosing served as a safety measure to reduce the risk of hypoglycemia. While it cannot be entirely ruled out that this might influence drug absorption, using the same administration schedule in both treatment periods means that any potential effect would be non-differential and unlikely to bias the pharmacokinetic comparison. Additionally, dapagliflozin's pharmacokinetics are not significantly affected by food, and for pioglitazone, food slightly delays absorption but does not impact AUC [10,11]. Consequently, administering glucose is unlikely to have affected the study's conclusion of bioequivalence.

The findings of this study support the pharmacokinetic comparability of the single-tablet formulation to the co-administration of the individual reference products under fasting conditions. This combination addresses complementary pathophysiological mechanisms in T2D by promoting insulin-independent glucose excretion and improving insulin sensitivity. Cardiovascular outcome trials and meta-analyses have shown that SGLT2 inhibitors are associated with a reduced risk of hospitalization for heart failure, potentially through hemodynamic and renal mechanisms. The evidence for pioglitazone is more nuanced, with some studies suggesting vascular benefits in selected populations, alongside known risks such as fluid retention. The natriuretic effects of SGLT2 inhibitors may theoretically counterbalance the fluid retention associated with thiazolidinediones [12,13]. However, this remains a mechanistic consideration and was not evaluated in the present study. Therefore, any potential cardiovascular or clinical complementarity between dapagliflozin and pioglitazone should be interpreted as a literature-based rationale only, not as a conclusion of this bioequivalence study. 

Consistent with this mechanistic rationale, a growing body of evidence supports the combined use of dapagliflozin and pioglitazone. The PRO-1 study demonstrated that FDC was non-inferior to separate administration in improving glycemic parameters and was well-tolerated [7]. Systematic reviews and meta-analyses have indicated favorable metabolic outcomes, whereas real-world data have suggested potential cardiovascular benefits [14-16]. Together, these findings reinforce the clinical rationale for combining these agents in appropriate patient populations.

Although the present study did not assess clinical efficacy or long-term outcomes, it provides pharmacokinetic evidence supporting the use of FDCs when concomitant therapy is indicated. From a regulatory standpoint, demonstrating bioequivalence under fasting conditions establishes pharmacokinetic equivalence between the FDC and co-administration under the standard bioequivalence framework. These findings support the use of FDC as an interchangeable formulation, providing a basis for approval and ensuring that its pharmacokinetic performance is consistent with that of the reference products. Additionally, FDCs may reduce pill burden and simplify treatment regimens, a particularly relevant benefit for long-term disease management.

Limitations

This study has several limitations that should be considered when interpreting the findings. First, the study was conducted in healthy adult male volunteers. This is because, in a crossover bioequivalence study, such a design is preferred to reduce variability and increase sensitivity to formulation-related differences. The use of only male subjects makes it difficult to generalize the results of this study to females, thereby preventing comparisons that could reveal potential gender-related differences in pharmacokinetics, such as hormone-related effects. Consequently, any extrapolations from the present findings concerning female patients should be done cautiously. Second, the pharmacokinetics were evaluated under single-dose, fasting conditions; therefore, no information on the drug's exposure under fed conditions or at steady state during prolonged intake was provided. Finally, because this study aimed to determine the relative bioavailability and bioequivalence of the two drug formulations, no information on their efficacy or safety was obtained. Moreover, this study was not registered with the Clinical Trials Registry of India, which may limit transparency and prospective traceability of the study protocol.

Nonetheless, these aspects are methodologically justified for evaluating the drug's equivalence in accordance with regulatory standards. Cross-over trials involving healthy volunteers are regarded as the gold standard for comparing pharmacokinetics, offering minimal variability and precise relative exposure measurement. Consequently, these limitations do not affect the validity of the bioequivalence results for the FDC under study.

## Conclusions

The FDC of dapagliflozin and pioglitazone was bioequivalent to the individual components in healthy adult male subjects under fasting conditions. The comparable rates and extents of exposure for both analytes indicate that co-formulation does not alter systemic availability and yields pharmacokinetic profiles equivalent to those of the individual components administered separately. The study treatments were well tolerated, and no serious or unexpected safety issues were observed. These results support the use of FDC as a pharmacokinetically suitable and convenient alternative to the coadministration of the individual drugs. Further studies may complement these findings by evaluating the pharmacokinetics under fed conditions, at steady state, and in patients with T2D.

## References

[1] Cho YK, Kim KS, Lee BW (2024). Efficacy and safety of pioglitazone add-on in patients with type 2 diabetes mellitus inadequately controlled with metformin and dapagliflozin: a multicenter, randomized, double-blind, and placebo-controlled study. Clin Ther.

[2] Sun R, Yuan L, Shen Y, Shen Z, Ding B, Ma J (2023). Impact of fixed combination of metformin and pioglitazone on insulin resistance of patients with type 2 diabetes: results of a randomized open-label study. Diabetes Metab Syndr Obes.

[3] Maksud N, Bera S, Naim MJ (2024). Dapagliflozin: a new hope for the therapeutic treatment of type 2 diabetes mellitus. Eur J Med Chem Rep.

[4] de Pablos-Velasco P (2010). Pioglitazone: beyond glucose control. Expert Rev Cardiovasc Ther.

[5] Mitra A, Wu Y (2012). Challenges and opportunities in achieving bioequivalence for fixed-dose combination products. AAPS J.

[6] Kalra S, Bhattacharya S (2022). Bioequivalence study of two different dapagliflozin tablet formulations in healthy adult Indian volunteers. J Diabetol.

[7] Singh AK, Seshadri KG, Unnikrishnan AG (2026). Efficacy and safety of fixed-dose dapagliflozin-pioglitazone in Indian adults with type 2 diabetes: results from the randomized phase 3 PRO-1 trial. Diabetol Metab Syndr.

[8] Eckland DA, Danhof M (2000). Clinical pharmacokinetics of pioglitazone. Experiment Clin Endocrinol Diabet.

[9] Cada DJ, Levien TL, Baker DE (2014). Dapagliflozin. Hosp Pharm.

[10] Dhillon S (2019). Dapagliflozin: a review in type 2 diabetes. Drugs.

[11] Waugh J, Keating GM, Plosker GL, Easthope S, Robinson DM (2006). Pioglitazone: a review of its use in type 2 diabetes mellitus. Drugs.

[12] Khan U, Majeed Z, Khan MH (2025). Efficacy and safety of pioglitazone add-on in patients with type 2 diabetes mellitus inadequately controlled with metformin and dapagliflozin: a systematic review and meta-analysis of randomised controlled trials. Endocrinol Diabetes Metab.

[13] Hong JH, Han KA, Hwang YC (2026). Efficacy and safety of high-dose pioglitazone as add-on therapy in patients with type 2 diabetes mellitus inadequately controlled with dapagliflozin and metformin: double-blind, randomized, placebo-controlled trial. Diabetes Metab J.

[14] Heo JH, Han KA, Hong JH (2024). Pioglitazone as add-on therapy in patients with type 2 diabetes mellitus inadequately controlled with dapagliflozin and metformin: double-blind, randomized, placebo-controlled trial. Diabetes Metab J.

[15] Anson M, Henney AE, Zhao SS (2024). Effect of combination pioglitazone with sodium-glucose cotransporter-2 inhibitors or glucagon-like peptide-1 receptor agonists on outcomes in type 2 diabetes: a systematic review, meta-analysis, and real-world study from an international federated database. Diabetes Obes Metab.

[16] Lo SC, Kornelius E, Liao PL, Huang JY, Yang YS, Huang CN (2023). Pioglitazone, SGLT2 inhibitors and their combination for primary prevention of cardiovascular disease and heart failure in type 2 diabetes: real-world evidence from a nationwide cohort database. Diabetes Res Clin Pract.

